# The effect of cognitive reserve on the cognitive connectome in healthy ageing

**DOI:** 10.1007/s11357-024-01328-4

**Published:** 2024-08-29

**Authors:** Annegret Habich, Eloy Garcia-Cabello, Chiara Abbatantuono, Lissett Gonzalez-Burgos, Paolo Taurisano, Thomas Dierks, José Barroso, Daniel Ferreira

**Affiliations:** 1 Division of Clinical Geriatrics, Center for Alzheimer Research, Department of Neurobiology, Care Sciences and Society, Karolinska Institute, Stockholm, Sweden; 2 University Hospital of Psychiatry and Psychotherapy, University of Bern, Bern, Switzerland; 3 Facultad de Ciencias de La Salud, Universidad Fernando Pessoa Canarias, Las Palmas, Spain; 4 University of Bari Aldo Moro, Bari, Italy; 5 Department of Clinical Psychology, Psychobiology and Methodology, Faculty of Psychology, University of La Laguna, La Laguna, Spain

**Keywords:** Cognitive network, Cognitive reserve, Ageing, Graph theory, Network analysis

## Abstract

During ageing, different cognitive functions decline at different rates. Additionally, cognitive reserve may influence inter-individual variability in age-related cognitive decline. These complex relationships can be studied by constructing a so-called cognitive connectome and characterising it with advanced graph-theoretical network analyses. This study examined the effect of cognitive reserve on the cognitive connectome across age. A total of 334 cognitively healthy participants were stratified into early middle age (37–50 years; n = 110), late middle age (51–64 years; n = 106), and elderly (65–78 years; n = 118) groups. Within each age group, individuals were subdivided into high and low cognitive reserve. For each subgroup, a cognitive connectome was constructed based on correlations between 47 cognitive variables. Applying graph theory, different global network measures were compared between the groups. Graph-theoretical network analyses revealed that individuals with high cognitive reserve were characterized by a stable cognitive connectome across age groups. High cognitive reserve groups only differed in modularity. In contrast, individuals with low cognitive reserve showed a marked reconfiguration of cognitive connectomes across age groups with differences extending over a variety of network measures including network strength, global efficiency, modularity, and small-worldness. Our results suggest a stabilizing effect of cognitive reserve on the cognitive connectome. Gaining further insights into these findings and underlying mechanisms will contribute to our understanding of age-related cognitive decline and guide the development of strategies to preserve cognitive function in ageing.

## Introduction

As the global population ages, the incidence of neurodegenerative diseases is expected to rise, calling for advancing our understanding of cognitive decline in older adults as the basis for its early detection [1]. However, cognitive trajectories in older adults are heterogeneous with different biological and environmental factors influencing their course [2, 3]. Importantly, some individuals show greater resistance to age-related cognitive decline, keeping cognitive functioning relatively preserved during the ageing progress [4, 5].

The resistance to age-related cognitive decline may partly be explained by cognitive reserve, which describes the ability to cope with cognitive deterioration due to age or neurodegenerative diseases through protective factors such as high education, crystallized intelligence, occupational attainment, or social and leisure activities [6]. Neuroimaging studies have shown that high cognitive reserve is associated with increased connectivity in dorsal attention and frontal-parietal control networks [7], indicating high neural and cognitive efficiency [8]. Further, cognitive reserve is considered to reflect enhanced brain network recruitment or the activation of alternative brain areas to compensate for cognitive impairment [9].

Apart from inter-individual differences in rates of cognitive decline during ageing, different cognitive functions within an individual also deteriorate at different rates [3, 10, 11]. Likewise, cognitive reserve impacts distinct cognitive functions and their decline in a differential manner [12, 13]. To understand the complex relationships between cognitive functions as well as individual cognitive variables, multivariate methods for data analyses are needed. In that context, graph theoretical analyses of cognitive data allow the investigation of the complex interactions between cognitive functions, i.e., the cognitive connectome. The great potential of graph-theoretical analyses to increase our understanding of cognitive ageing has been demonstrated by several recent studies [14–18]. In a previous study, we found age-related differences in the cognitive connectome [15]. We showed increased correlations between cognitive variables in late middle age that we interpreted as a compensatory mechanism to maintain cognitive performance. In contrast, we observed a reduction in correlations in the elderly group that was associated with an aberrant organization of the cognitive connectome and lower cognitive performance. However, our previous study could not directly demonstrate a compensatory mechanism, and thus this interpretation remained speculative.

Hence, the present study aimed to elucidate a potential compensatory mechanism underlying the age-related connectome differences reported in the previous study [15]. Since cognitive reserve has been closely related to compensatory mechanisms [8, 9], we investigated whether cognitive reserve influences properties of the cognitive connectome with increasing age. The current study had three aims. First, we investigated the interaction between age and cognitive reserve on cognitive performance to test whether high cognitive reserve reduces the negative effect of ageing on cognitive performance. Second, we compared cognitive connectomes across age groups separately in high and low cognitive reserve groups. We hypothesized an increasing covariance in the cognitive connectome with increasing age in individuals with high cognitive reserve, delineating a compensatory mechanism, while we expected a less pronounced covariance with increasing age in individuals with low cognitive reserve. Third, we compared cognitive connectomes in high versus low cognitive reserve groups in three different age groups. We hypothesized that individuals with a high cognitive reserve would show a higher covariance in comparison to individuals with a low cognitive reserve, and that these differences would be larger in older groups, delineating a compensatory mechanism.

## Materials and methods

### Participants

The sample consisted of 334 participants from the GENIC (Group of Neuropsychological Studies of the Canary Islands) database [11]. All participants were native Spanish speakers recruited from the Canary Islands in Spain, aged 37 to 78 years. Sex was evenly distributed across ages by design.

Methods followed the procedures described by Garcia-Cabello and colleagues [15]. In short, we included volunteers based on 5 eligibility criteria: (1) absence of dementia according to Mini-Mental State Examination (MMSE) ≥ 24; Blessed Dementia Rating Scale (BDRS) < 4; and Functional Activities Questionnaire (FAQ) < 6; (2) absence of mild cognitive impairment (MCI) based on the clinical judgement made by two neuropsychologists applying Winblad et al. criteria [19] to age-, sex-, and education-corrected scores from the administration of a comprehensive cognitive battery (detailed in Ferreira et al., 2015 [20]); (3) right handedness assessed through the Edinburgh Handedness Inventory; (4) absence of stroke, tumours, and/or hippocampal sclerosis in MRI, according to an expert radiologist when available; (5) absence of neurological or psychiatric disorders, history of substance abuse, and/or systemic health conditions impacting neuropsychological functioning. An exception was made for the BDRS. Although the clinical cut-off for the BDRS scale is often established at $\ge$4 points, [21, 22] the ‘‘changes in personality, interests, and drive’’ subscale may influence the BDRS total score and does not necessarily reflect functional impairment. As we aimed to exclude only those individuals who had a functional impairment, we included participants with total BDRS scores $\ge$4 (n = 15) if: (a) ≥ 70% of the BDRS total score resulted from the ‘‘changes in personality, interests and drive’’ subscale; and (b) a score $\le$1.5 was obtained in the other two subscales (‘‘changes in performance of everyday activities’’ and ‘‘changes in habits’’). The same approach has been applied in previous studies [14, 15, 23].

All included participants provided their written informed consent before entering the study in accordance with the Declaration of Helsinki. The research protocol was approved by the local ethics committee of the University of La Laguna (Spain).

### Cognitive reserve and cognitive assessment

In this study, we used the Information subtest from the Wechsler Adult Intelligence Scale (WAIS-III; [24]) as a crystallized intelligence proxy of cognitive reserve. This choice was based on a previous study in the same cohort showing that the WAIS-III Information subtest consistently correlated with other proxies of cognitive reserve and outperformed them in mediating the relationship between cortical thickness and cognitive performance [25]. Participants’ global cognitive performance was assessed using the MMSE [26]. In addition, all participants underwent a comprehensive neuropsychological assessment to characterize patients’ cognitive profile. The 47 cognitive variables derived from the assessment are reported in Table 1. For cohort characterization (Table 2) and interpretation of results, all cognitive variables were allocated to five cognitive modules, i.e. (i) verbal memory, (ii) visual memory and visuospatial functions, (iii) executive and premotor functions, (iv) procedural memory, and (v) processing speed (Table 1). The allocation to cognitive modules was based on a modular analysis using the Newman algorithm [27] from our previous study [15]. For network analyses, all 47 variables were converted to z-scores using means and standard deviations of the whole cohort. To ensure a better interpretability of all analysis results, higher z-scores denoted better performance in all variables and modules, including procedural memory and processing speed, whose cognitive variables were inverted. The performance in each cognitive module was derived by averaging z-scores of cognitive variables included in the distinct modules.

**Table 1:** Overview of cognitive tests

Test name	Cognitive variables	Corresponding cognitive module
Logical Memory (LM; Wechslser, 1997b)	LM A immediate	Verbal memory (VM)
	LM B1 immediate	
	LM B2 immediate	
	LM A delayed	
	LM B delayed	
	LM A recognition	
	LM B recognition	
*Test de Aprendizaje Verbal España-Complutense* (TAVEC, Spanish Version of California Verbal Learning Test; Benedet & Alejandre, 1998)	TAVEC 1st trial	
	TAVEC learning	
	TAVEC interference	
	TAVEC immediate total	
	TAVEC cued immediate recall	
	TAVEC delayed total	
	TAVEC cued delayed recall	
Facial Recognition Test (FRT; Benton et al., 1983)	FRT	Visual memory and visuospatial functions (VMV)
Judgment of Line Orientation Test (JLOT; Benton et al., 1983)	JLOT 1st half	
	JLOT 2nd half	
Spatial span backward	Spatial span backward	
Visual Reproduction (VR; Wechsler, 1997b)	VR I total	
	VR II total	
	VR copy	
	VR recognition	
Block Design; Wechsler (1997a)	Block design total	
Boston Naming Test (BNT; Kaplan et al., 1983)	BNT	
8/30 Spatial Recall Test (8/30 SRT); Modification of the 7/24 SRT; Rao et al., 1984	8/30 1st trial	
	8/30 learning	
	8/30 interference	
	8/30 immediate	
	8/30 delayed	
Stroop Test (STROOP; Golden, 1978)	STROOP words	Executive and premotor functions (EPF)
	STROOP colours	
	STROOP inhibition	
Verbal fluency; Benton, Hamsher & Sivan, 1989 (letters and animals); Piatt et al., 1999 (actions)	Phonetic fluency	
	Semantic fluency	
	Action fluency	
Digit span forward	Digit span forward	
Digit span backward	Digit span backward	
Spatial span forward	Spatial span forward	
Luria’s Premotor Functions, Hand Alternative Movements (HAM)	HAM right	
	HAM left	
	HAM motor coordination	
Hanoi Tower (HT; Simon, 1975)	HT 1st trial	Procedural memory (PM)
	HT learning	
	HT long delay	
PC-Vienna System (PCV; Schuhfried, 1992)	PCV decision time	Processing speed (PS)
	PCV motor time	
Color Trails Test (CTT; D’Elia and Satz, 1989)	CTT part A

**Table 2:** Characteristics of participants

	High cognitive reserve				Low cognitive reserve		
	Early middle age (37–50 yrs)	Late middle age (51 – 64 yrs)	Elderly (65–78 yrs)	Early middle age (37–50 yrs)		Late middle age (51 – 64 yrs)	Elderly (65–78 yrs)
# participants	59	67	40	51		39	78
# women (%)	22 (37.3%)	32 (47.8%)	17 (42.5%)	36 (70.6%)		31 (79.5%)	50 (64.1%)
Age	45.8 (3.3)	58.1 (4.0)	70.3 (3.8)	44.6 (3.5)		58.6 (4.8)	70.5 (3.9)
Educative level							
Illiteracy	0	0	0	0		0	4
Unfinished primary studies	0	0	2	2		6	33
Completed primary studies	11	10	9	37		21	32
Completed secondary studies	27	13	11	7		7	8
University studies	21	44	18	5		5	1
MMSE	29.1 (1.0)	28.9 (1.1)	28.6 (1.5)	28.9 (1.5)		28.1 (1.6)	27.6 (1.5)
WAIS III Info	20.3 (2.8)	21.9 (2.6)	20.1 (3.1)	10.5 (3.1)		10.1 (3.3)	9.3 (2.9)
Verbal memory	0.5 (0.5)	0.4 (0.6)	-0.1 (0.6)	0.1 (0.6)		-0.1 (0.6)	-0.7 (0.6)
Visual memory and visuospatial functions	0.6 (0.3)	0.3 (0.4)	0.0 (0.4)	0.2 (0.4)		-0.2 (0.6)	-0.8 (0.5)
Executive and premotor functions	0.6 (0.5)	0.5 (0.5)	0.0 (0.6)	0.1 (0.4)		-0.3 (0.7)	-0.8 (0.4)
Procedural memory	-0.2 (0.6)	0.0 (0.7)	-0.3 (0.6)	0.2 (0.8)		0.2 (1.0)	-0.0 (0.8)
Processing speed	-0.5 (0.4)	-0.4 (0.4)	0.2 (0.6)	-0.5 (0.5)		0.1 (0.6)	0.9 (0.7)

### Cognitive connectomes and network analysis

We constructed cognitive connectomes from the 47 cognitive variables after z-scoring and inversion of z-scores for procedural memory and processing speed variables (Table 1). Pearson’s correlation coefficients were calculated between each pair of cognitive variables. Considering the non-uniform decline of cognitive functions during the ageing process and potential differences between reserve groups, we refrained from thresholding correlations based on p-values to avoid the introduction of artificial group differences. Negative correlations and self-connections were removed from the connectomes.

To evaluate network topologies, we mainly selected global measures of centrality, integration, and segregation that were reported to be stable in a previous study [28]. Specifically, we calculated network strength (measure of centrality, average of nodal correlation strengths), global efficiency (measure of integration, inverse of the average shortest path between all nodes in the network), local efficiency (measure of segregation, inverse of the average shortest path between all neighbours of a node), modularity (measure of integration and segregation, extent to which a network can be separated into distinct modules), and small-worldness (measure of integration and segregation considering the clustering coefficient relative to the average shortest path length of a network [29]). Network strength was calculated on the weighted correlation matrices. All other graph measures were calculated on binarized correlation matrices across network densities between 20 to 60%, in steps of 1%. This density range was selected based on the entire cohort to exclude disconnected (densities < 20%) and random network topologies (densities > 60% with small-worldness < 1).

### Statistical analysis

Group differences in demographic variables were assessed using ANOVAs and χ^2^ for continuous and categorical variables respectively. Correlations between variables were tested with Spearman correlations.

To test aim 1 and investigate the interaction between age and cognitive reserve, we applied a 2-way MANOVA. Participants were stratified into three age groups (i.e., early middle age, 37—50 years; late middle age 51—64 years; and elderly 65—78 years). Each age group was further split into two levels of cognitive reserve based on the median of the cohort in the WAIS-III Information subtest as a crystallized intelligence proxy of cognitive reserve (i.e., high cognitive reserve, > 15; low cognitive reserve, ≤ 15). Age group (early middle age, late middle age, elderly) and level of cognitive reserve were entered as between-subject factors. Performances in the five cognitive modules, i.e., verbal memory, visual memory and visuospatial functions, executive and premotor functions, procedural memory, and processing speed, were included as dependent variables. To gain further understanding on which specific cognitive modules are influenced by age and cognitive reserve, we tested for the interaction between age and cognitive reserve separately for each five cognitive module using ANOVAs with age group and cognitive reserve as between-subject factors. As a sensitivity analysis, we followed up on the main MANOVA with a new model adding sex as an additional independent variable. In this second MANOVA we focussed on the three-way interaction between age, cognitive reserve, and sex, to test whether sex influenced the results from our main MANOVA.

For aims 2 and 3, differences in network measures between age and cognitive reserve groups were assessed using 1′000 non-parametric permutations in the previously defined range of network densities (20% to 60%, ins steps of 1%). For the small-worldness measure, differences between age and cognitive reserve groups were assessed using 100 non-parametric permutations across the range of network densities. Global network measures that showed significant differences in ≥ 10 network densities were considered significant, to focus on stable differences across the range of densities [30]. All network analyses were carried out in Matlab, using *BRAPH v. 1.0.0*. [31]. A *p*-value < 0.05 (two-tailed) was deemed significant in all analyses.

## Results

The key demographic characteristics of the six groups are displayed in Table 2. Age and performance in the WAIS-III Information subtest differed between groups by design. Additionally, groups differed in MMSE and educative level, since both respectively correlated with age (*r*_*S*_ = -0.33, *p* < 0.001) and WAIS-III Information (*r*_*S*_ = 0.7, *p* < 0.001), as expected in our cohort. The groups also differed in sex distribution.

### High cognitive reserve attenuates the age-related effect on cognitive performance

**Fig. 1:**
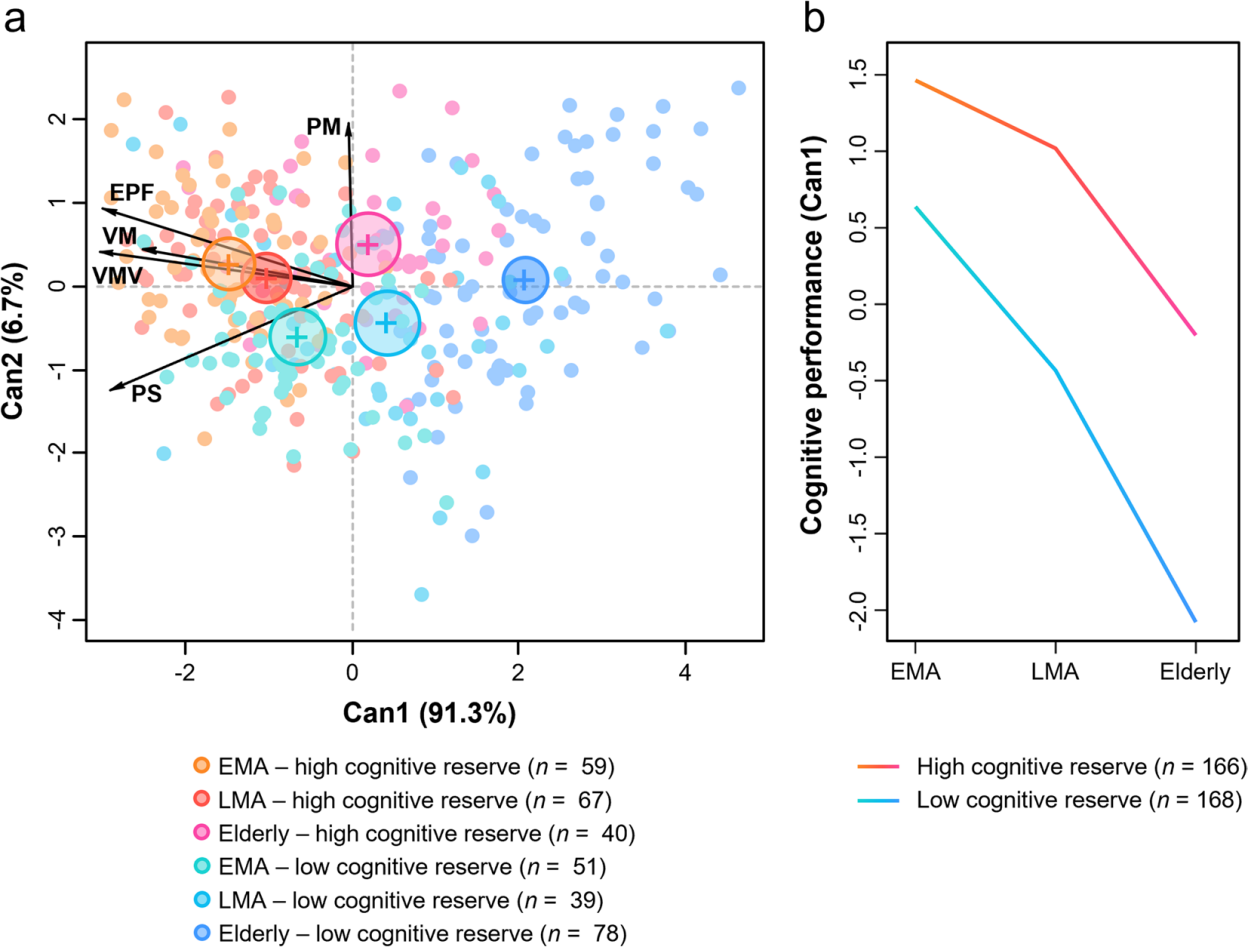
**a** Interaction between age and cognitive reserve obtained from MANOVA. Axes represent the canonical discriminant functions 1 and 2. Since only the canonical function 1 was statistically significant, results are interpreted horizontally along the x-axis. Large circles represent the means of each group. Arrows represent the cognitive modules used in the MANOVA. **b** Means of canonical function 1 (*F*_(10, 654)_ = 2.248, *p*_(age*cognitive reserve)_ = 0.014) from MANOVA for the six groups. In Panel B, means of canonical function 1 were inverted with respect to Panel A so that positive values represent higher cognitive performance. EMA = Early middle age, EPF = Executive and premotor functions, LMA = Late middle age, PM = Procedural memory, PS = Processing speed, VM = Verbal memory, VMV = Visual memory and visuospatial functions

Our main MANOVA showed a statistically significant interaction effect between age and cognitive reserve (Wilks = 0.93; F_(10.648)_ = 2.23; p < 0.05) on cognitive performance represented by the five cognitive modules (i.e., verbal memory, visual memory and visuospatial functions, executive and premotor functions, procedural memory, and processing speed). Overall, we observed smaller age differences in cognitive performance in the high reserve group as compared with the low reserve group (Fig. 1 A & B). Follow-up ANOVAs showed statistically significant interaction effects between age and cognitive reserve in processing speed (*F* = 8.59, *p* < 0.001), visual memory and visuospatial functions (*F* = 5.00, *p* < 0.01), and executive and premotor functions (*F* = 3.25, *p* = 0.04), again, with smaller age differences in these cognitive modules in the high reserve group as compared with the low reserve group. Since our groups showed differences in sex distribution, we performed a sensitivity analysis for the MANOVA including sex as an additional independent variable. We tested for the three-way interaction between age, cognitive reserve, and sex. That interaction was not statistically significant (Wilks = 0.97; F_(10.636)_ = 0.957; p = 0.48), suggesting that the difference in sex distribution across groups did not influence the results from our main MANOVA.

### High cognitive reserve stabilizes the cognitive connectome during ageing

To address our second aim, we compared cognitive connectomes across age groups with high and low cognitive reserve.

**Fig. 2:**
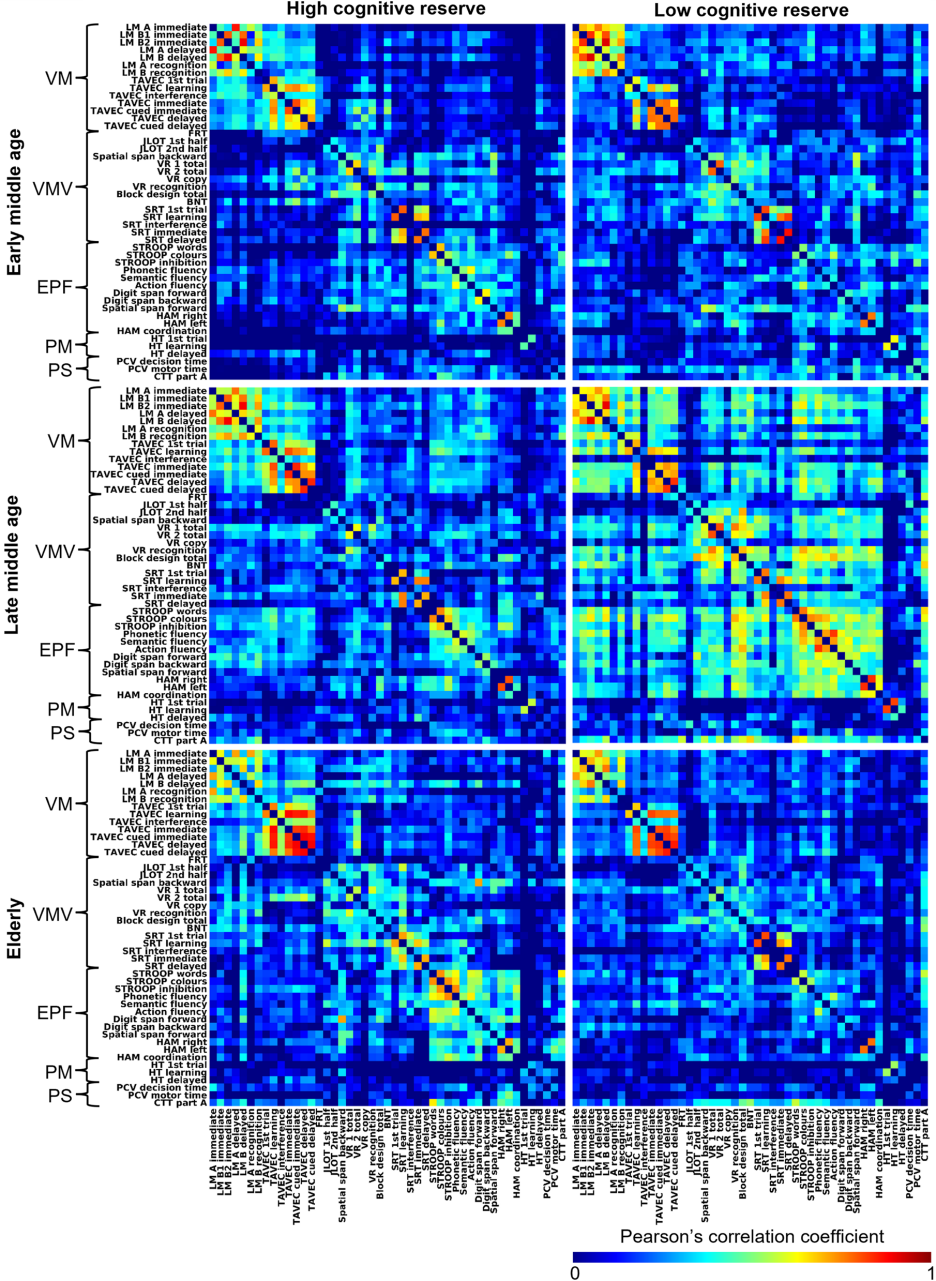
Weighted correlation matrices across age and cognitive reserve groups with module affiliation of cognitive variables denoted with brackets. BNT = Boston Naming Test, CTT = Color Trails Test, EPF = Executive and premotor functions, FRT = Facial Recognition Test, HT = Hanoi Tower, JLOT = Judgment of Line Orientation Test LM = Logical Memory (Text Subtest I and II, WMS-III), PCV = PC-Vienna System, PM = Procedural memory, PS = Processing speed, VM = Verbal memory, VMV = Visual memory and visuospatial functions, VR = Visual Reproduction (Drawing Subtest I and II, WMS-III)

Figure 2 shows correlation matrices featuring the five cognitive modules previously described by Garcia-Cabello and colleagues [15]. Visual inspection of the correlation matrices across age groups revealed a relative preservation of the cognitive connectome in high cognitive reserve groups with similar patterns of balanced intra- and inter-module correlations. In contrast, in low cognitive reserve, the cognitive connectome undergoes more drastic restructuring in the ageing process. Specifically, an increase in correlation strength can be observed in late middle age compared with early middle age and elderly, spanning both intra- and inter-module correlations. In elderly, both intra- and inter-module correlations decrease drastically compared with late middle age.

**Fig. 3:**
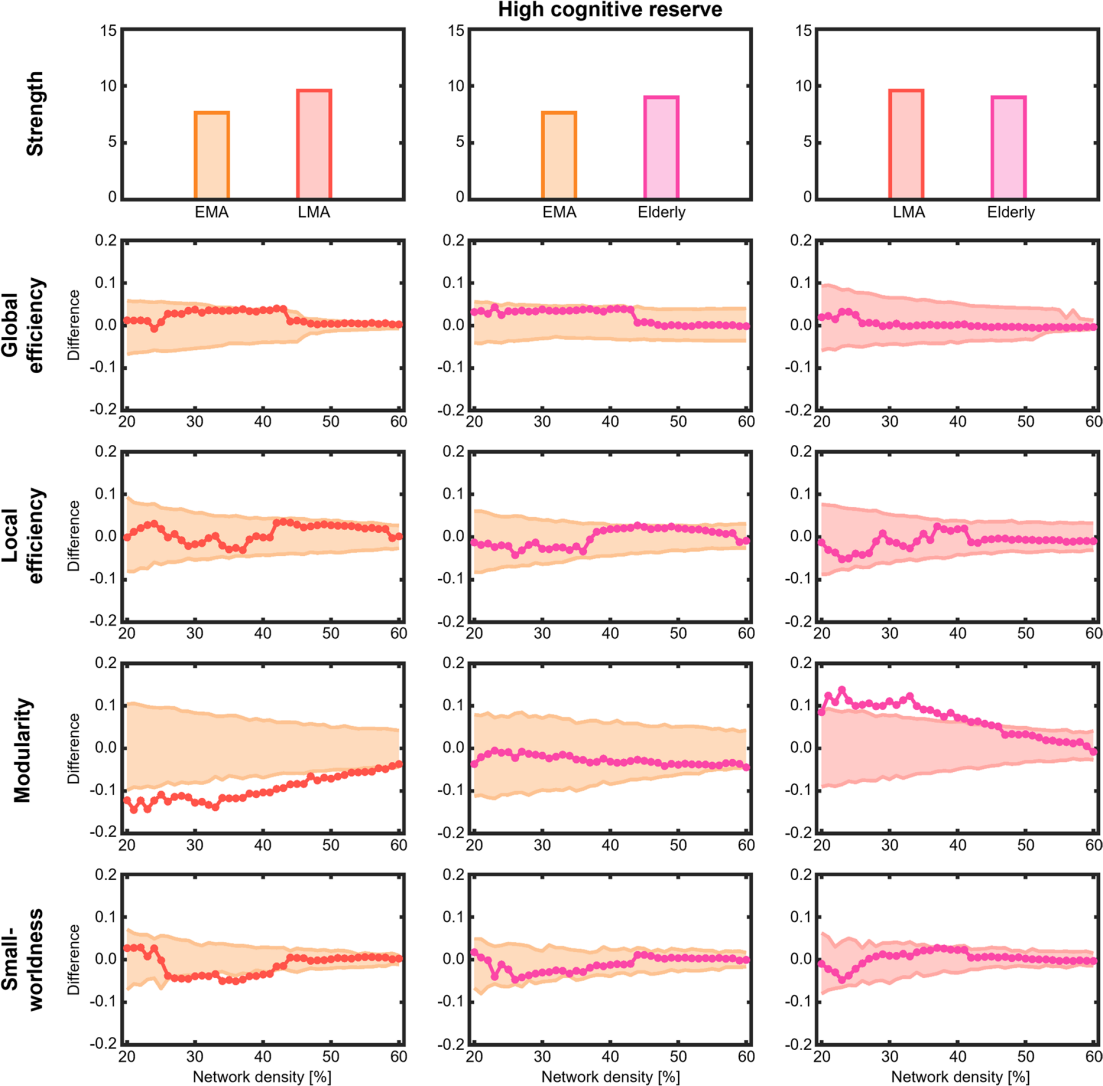
Comparison of global network measures across age groups with high cognitive reserve. Network densities are displayed on the x-axis, ranging from min = 20% to max = 60%, in 1% steps. Differences between age groups are shown on the y-axis. Differences between groups are significant when the circles are outside of the shaded area. EMA = early middle age, LMA = late middle age

These findings were supported by comparisons of network measures across age groups with differences being more restricted in high cognitive reserve and more extensive in low cognitive reserve. Specifically, in individuals with high cognitive reserve, the late middle age group exhibited a lower modularity and lower small-worldness compared with the early middle age group (Fig. 3). The elderly group showed a higher modularity compared with the late middle age group. No significant differences were observed in the remaining network measures. Further, no differences emerged between the elderly and early middle age groups in any of the network measures.

**Fig. 4:**
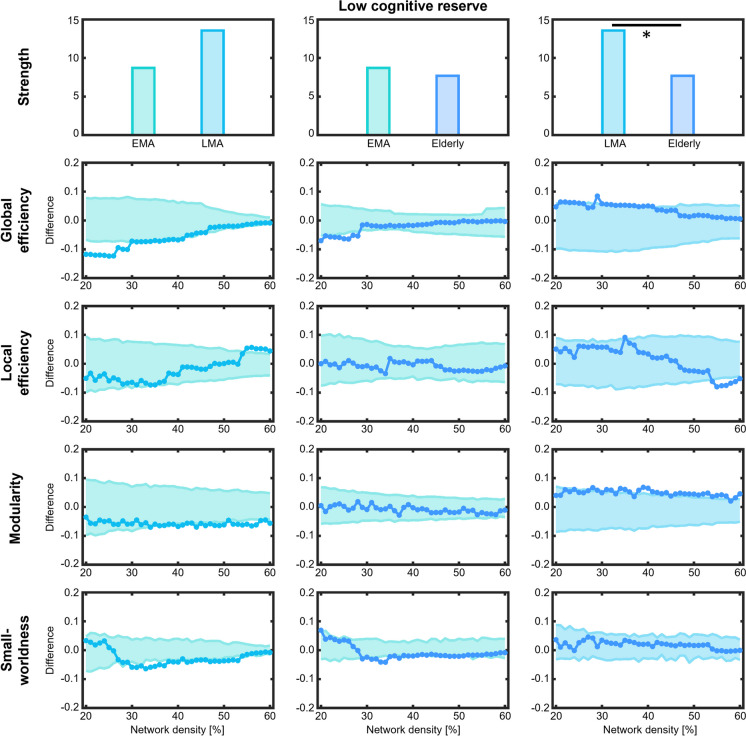
Comparison of global network measures across age groups with low cognitive reserve**.** Network densities are displayed on the x-axis, ranging from min = 20% to max = 60%, in 1% steps. Differences between age groups are shown on the y-axis. Differences between groups are significant when the circles are outside of the shaded area. EMA = early middle age, LMA = late middle age

In individuals with low cognitive reserve, the late middle age group showed lower global efficiency, lower modularity, and lower small-worldness compared with the early middle age group (Fig. 4). The elderly group showed lower small-worldness compared with the early middle age group. Additionally, the elderly group exhibited lower network strength, higher global efficiency, and higher modularity compared with the late middle age group.

### Cognitive connectomes in high and low cognitive reserve are most different in late middle age

**Fig. 5:**
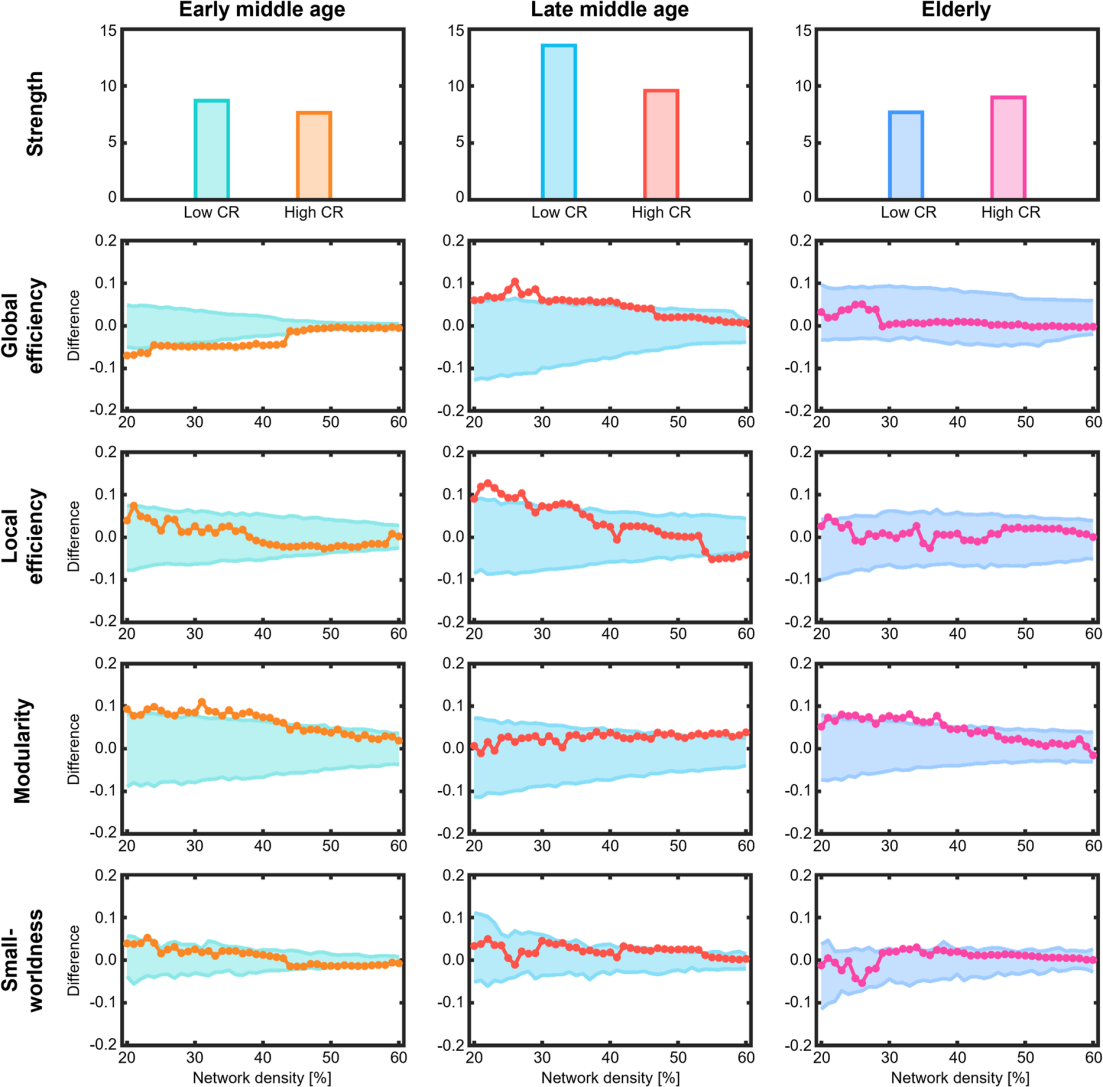
Comparison of global network measures between cognitive reserve groups. Network densities are displayed on the x-axis, ranging from min = 20% to max = 60%, in 1% steps. Differences between age groups are shown on the y-axis. Differences between groups are significant when the circles are outside of the shaded area. CR = cognitive reserve

To address our third aim, we compared cognitive connectomes between high and low cognitive reserve within age groups (Fig. 5).

On visual inspection (Fig. 2), in early middle age, individuals with high cognitive reserve showed more intra-module correlations and less inter-module correlations compared with the low cognitive reserve group. However, the most striking differences presented in late middle age, with overall lower covariance in the high cognitive reserve group compared with the low cognitive reserve group. Finally, elderly individuals with high cognitive reserve showed more intra-module correlations compared with their low cognitive reserve counterparts.

Similar results emerged when comparing network measures across cognitive reserve groups. Regarding the early middle age group, the high cognitive reserve group showed a significantly lower global efficiency and higher modularity compared with the low cognitive reserve group. In late middle age, the high cognitive reserve group showed a significantly higher global and local efficiency compared with the low cognitive reserve group. In the elderly group, the high cognitive reserve group showed higher modularity compared with the low cognitive reserve group.

## Discussion

While previous studies revealed the impact of healthy and pathological ageing on the cognitive connectome [14–18], this is the first study to determine the impact of high and low cognitive reserve on cognitive connectomes during the ageing process.

For the first aim in our study, we demonstrated an interaction effect of cognitive reserve and age on global cognitive performance, with high cognitive reserve attenuating age-related cognitive decline. This protective effect of cognitive reserve on age-related cognitive decline is well-established in the literature [32]. Considering distinct cognitive modules, the interaction effect between cognitive reserve and cognition during ageing spanned visual memory and visuospatial memory, executive-premotor functions, and processing speed. Therein, high performance in visual memory and visuospatial functions as well as executive premotor functions characterised early and late middle age individuals with high cognitive reserve and, to a lesser degree, the elderly group with high cognitive reserve and early middle age individuals with low cognitive reserve. In contrast, worse performance in all cognitive modules except for procedural memory characterised the elderly group with low cognitive reserve. This finding confirms the importance of visuospatial and executive premotor functions during successful ageing as well as the contribution of processing speed to cognitive ageing [33–35].

To further disentangle the effect of cognitive reserve and age on the cognitive connectome, we investigated the effect of age separately in high and low cognitive reserve. In a previous study, in which we identified age-specific cognitive connectomes irrespective of cognitive reserve, we surmised that the observed higher integration of cognitive modules in late-middle age reflected a compensatory mechanism in the face of cognitive decline [15]. As such, in the current study, we hypothesized that the cognitive connectome in groups with high cognitive reserve would reflect a recruitment of cognitive functions across cognitive modules, resulting in increasingly more integrated connectomes, to counter age-related cognitive decline. In contrast, we hypothesized that groups with low cognitive reserve would remain more stable during the ageing process, reflecting their inefficiency in pooling cognitive resources to fulfil cognitive demands. These hypotheses were partly based on functional neuroimaging studies that demonstrated that an increased recruitment of frontal regions or additional contralateral hemispheric regions during task performance characterized individuals who preserved their cognitive function into old age [35–37]. This additional recruitment of brain regions and circuits was considered to occur if specialized resources to perform a cognitive task were insufficient [35, 37–39].

Our hypotheses were partially confirmed insofar as in individuals with high cognitive reserve, the late middle age group showed a slight increase in inter-module correlations compared with early middle age and elderly groups. This corresponds with the lower modularity observed in late middle age compared with early middle age and elderly groups as well as the lower small-worldness in late middle age compared with early middle age. The increase in inter-module correlations was particularly evident between verbal memory and executive and premotor modules. This is in line with the previously reported importance of executive functions for memory processes [40]. We found that in elderly with high cognitive reserve, modularity was higher compared with late middle age, driven by an increase in intra-module correlations and a decrease in inter-module correlations. This aligns with a highly selective recruitment of related cognitive resources, indicating the presence of adaptable and flexible processes to counter age-related cognitive decline connected to successful compensatory processes [4, 41]. However, contrary to our expectations, we found that individuals with high cognitive reserve showed a relatively stable cognitive connectome across age groups, especially within verbal memory and executive and premotor modules. The observed stability in the cognitive connectome of high cognitive reserve groups aligns with the stabilizing impact of cognitive reserve on cognitive performance that was previously reported to extend to individuals aged 70 + years [42]. In contrast, we found that the cognitive connectome of individuals with low cognitive reserve showed more prominent differences across age groups. Even intra-module correlations, specifically in verbal memory, visual memory and visuospatial functions, and executive and premotor modules showed a high variability. The most striking changes occurred in late middle age, which was characterized by an extensive unspecific increase in intra- and inter-module correlations compared with early middle age and elderly groups.

This higher integration of the cognitive connectome in late middle age was also confirmed in our quantitative analyses. While early middle age and elderly groups with low cognitive reserve only differed in small-worldness, the late middle age group exhibited lower global efficiency and lower modularity compared with early middle age and elderly groups. Additionally, the late middle age group showed lower small-worldness compared with the early middle age group and higher network strength compared with the elderly group. Taken together, these findings indicate a less segregated (more integrated) network topology arising in the late middle age group with low cognitive reserve. These changes align with neural dedifferentiation processes in late middle age that may suggest the recruitment of additional neural resources as a potential compensatory mechanism in the face of accelerated cognitive decline [43, 44]. However, as the observed dedifferentiation is predominantly associated with low cognitive reserve, which was characterized by lower performance across all cognitive modules in our ANOVAs, it may rather indicate an unsuccessful compensation process [41]. Moreover, the lower segregation (higher integration) of the cognitive connectome observed in late middle age might denote a global deterioration of cognitive functions irrespective of module affiliation. As such, our results align with previous observations of less specific and more integrated cognitive connectome in patients with mild cognitive impairment and Alzheimer’s disease in comparison with healthy elderly participants [16, 18]. Notwithstanding that all participants in this study were cognitively healthy, a “concordance of dysfunctions” as previously described for pathological ageing by Wright and colleagues [18], might also be applicable for low cognitive reserve groups. Additionally, the observed lower integration of the cognitive connectome in elderly with both high and low cognitive reserve, especially in comparison with late middle groups, may be related to increased interindividual variability as well increased intraindividual cognitive dispersion observed with increasing age [45–47].

Adding onto the finding of a more stable cognitive connectome during the ageing process in individuals with high cognitive reserve, we investigated the impact of cognitive reserve on the cognitive connectome at different ages. Comparing high and low cognitive reserve groups at different ages, we found differences in network topologies across all age groups. Notably, the modular structure with higher intra-module and lower inter-module correlations remained more intact in high cognitive reserve groups, especially in early middle age and elderly groups. This is also evident in the higher modularity exhibited by early middle age and elderly groups with high cognitive reserve compared with their low cognitive reserve counterparts. Additionally, the sparsity of inter-module correlations in the early middle age groups with high cognitive reserve was probably driving the lower global efficiency in this group compared with the early middle age group with low cognitive reserve. In late middle age, an increase in both intra- and inter-module correlations was particularly noticeable in the low cognitive reserve group. This difference was also reflected the higher global and local efficiency in the high cognitive reserve compared with the low cognitive reserve group, pointing out a more stable cognitive organization in terms of efficiency in the high cognitive reserve group. As stated above, this increase in correlations in the low cognitive reserve group potentially denotes a dedifferentiation of cognitive functions related to possible unsuccessful compensatory processes. The dedifferentiation process seems to be less evident in the high cognitive reserve group, indicating a more selective recruitment of more closely related cognitive functions i.e. functions belonging to the same module. Differences between cognitive reserve groups also emerged in the elderly, in so far as individuals with high cognitive reserve exhibited higher correlations, especially within modules, compared with individuals with low cognitive reserve in whom both intra- and inter-module correlations were substantially reduced. In terms of network measures, elderly with high cognitive reserve exhibited a higher modularity compared with elderly with low cognitive reserve. These findings suggest that elderly individuals with high cognitive reserve present a connectome where cognitive modules are more delimited from each other, including more specialized and potentially more successful compensatory processes restricted to closely related cognitive functions. In contrast, lacking the protective effect of cognitive reserve might have resulted in more heterogeneous trajectories of cognitive decline in elderly individuals with low cognitive reserve resulting in the observed weakly integrated cognitive connectome.

Some limitations should be noted. Firstly, we use the WAIS-III Information score as a proxy for cognitive reserve. In contrast to other cognitive reserve measures that incorporate information on education, occupation, or leisure activities, the WAIS-III Information score mainly assesses crystallized intelligence. However, we previously demonstrated that, among several proxies of cognitive and brain reserve, the WAIS-III Information score had the greatest capacity to attenuate the effect of reduced cortical thickness on cognition as well as predict cognitive performance, making it the preferred proxy of reserve in our cohort [25, 48]. Secondly, we did not include a measure of brain integrity in this study, and thus, cannot rule out its potential contribution to the observed effects of cognitive reserve on the cognitive connectome. Thirdly, there is currently no method to construct individual cognitive connectomes. Individual cognitive connectomes would have enabled us to model cognitive reserve and other demographic and clinical characteristics of participants as continuous variables, investigating their impact on the individuals’ cognitive connectomes in greater detail. Instead, we had to perform comparisons between group-based cognitive connectomes by differentiating individuals with high and low cognitive reserve with a median split procedure. While the median split is a standard procedure, it might have led to the inclusion of comparable individuals around the median split in both groups, therefore, potentially underestimating group differences. Fourthly, while sex was equally distributed across the three age groups by design, women were overrepresented in the groups with low cognitive reserve. Similarly, individuals with lower educational attainment were more likely to have low cognitive reserve, with particularly low education levels in the elderly group with low cognitive reserve. In turn, this led to the overrepresentation of men with high education level in the high cognitive reserve groups. However, we demonstrated that sex effects did not mediate the observed interaction between cognitive reserve and age on cognitive performance, and sex effects are supposedly diminishing with increasing age [49]. Hence, we consider a potential confounding effect of sex in our results to be very unlikely, especially in the older groups. Fifthly, the number of participants included in each of the six groups differed. More precisely, elderly with low cognitive reserve made up the largest group (*n* = 78), whereas late middle age with low cognitive reserve (*n* = 39) and elderly with high cognitive reserve (*n* = 40) were the smallest groups. Since the cognitive connectomes are based on Pearson’s correlation coefficients, network topologies are more stable at larger group sizes [50]. To curb the impact of random covariances in our analyses, we restricted our network analyses to non-random network topologies, across which the majority of group differences in global network measures were stable. Sixthly, network measures tend to produce more reliable results with increasing number of nodes. To account for this effect, we mainly selected network measures that were previously shown to be stable [28]. Additionally, the inclusion of 47 cognitive variables in our cohort more than doubled the number of cognitive variables included in previous studies of the cognitive connectome in ageing [16, 17]. Finally, the cross-sectional design of our study only allows for the comparison of age groups instead of providing insights into individual longitudinal trajectories across multiple cognitive domains [3]. A longitudinal approach would be particularly interesting to rule out potential generational effects in our cohort, insofar as elderly were more likely to have low cognitive reserve and a lower educational level.

To conclude, our study demonstrates that high cognitive reserve exerts a stabilizing influence on the cognitive connectome during the ageing process. In contrast, individuals with low cognitive reserve show more prominent differences in their cognitive connectome with increasing age. In our cohort, the most striking reorganization of the cognitive connectome occurred in late middle-aged individuals with low cognitive reserve, who exhibited a less segregated (more integrated) cognitive connectome. This suggests the presence of a cognitive dedifferentiation process in the face of global cognitive decline, either due to the unselective recruitment of cognitive resources or a concordant decline across several cognitive functions. Overall, gaining further insights into the influence of cognitive reserve on the associations between cognitive functions during ageing will contribute to a better understanding of age-related cognitive decline and foster the development of strategies to preserve cognitive function in ageing.

## Data Availability

The data supporting the study's findings are available for qualified researchers upon request. Requests should be directed to Daniel Ferreira, daniel.ferreira.padilla@ki.se.
